# Environmental Perturbation of the Circadian Clock Disrupts Pregnancy in the Mouse

**DOI:** 10.1371/journal.pone.0037668

**Published:** 2012-05-23

**Authors:** Keith C. Summa, Martha Hotz Vitaterna, Fred W. Turek

**Affiliations:** 1 Center for Sleep and Circadian Biology, Department of Neurobiology, Northwestern University, Evanston, Illinois, United States of America; 2 Northwestern University Feinberg School of Medicine, Chicago, Illinois, United States of America; Nagoya University, Japan

## Abstract

**Background:**

The circadian clock has been linked to reproduction at many levels in mammals. Epidemiological studies of female shift workers have reported increased rates of reproductive abnormalities and adverse pregnancy outcomes, although whether the cause is circadian disruption or another factor associated with shift work is unknown. Here we test whether environmental disruption of circadian rhythms, using repeated shifts of the light:dark (LD) cycle, adversely affects reproductive success in mice.

**Methodology/Principal Findings:**

Young adult female C57BL/6J (B6) mice were paired with B6 males until copulation was verified by visual identification of vaginal plug formation. Females were then randomly assigned to one of three groups: control, phase-delay or phase-advance. Controls remained on a constant 12-hr light:12-hr dark cycle, whereas phase-delayed and phase-advanced mice were subjected to 6-hr delays or advances in the LD cycle every 5–6 days, respectively. The number of copulations resulting in term pregnancies was determined. Control females had a full-term pregnancy success rate of 90% (11/12), which fell to 50% (9/18; p<0.1) in the phase-delay group and 22% (4/18; p<0.01) in the phase-advance group.

**Conclusions/Significance:**

Repeated shifting of the LD cycle, which disrupts endogenous circadian timekeeping, dramatically reduces pregnancy success in mice. Advances of the LD cycle have a greater negative impact on pregnancy outcomes and, in non-pregnant female mice, require longer for circadian re-entrainment, suggesting that the magnitude or duration of circadian misalignment may be related to the severity of the adverse impact on pregnancy. These results explicitly link disruptions of circadian entrainment to adverse pregnancy outcomes in mammals, which may have important implications for the reproductive health of female shift workers, women with circadian rhythm sleep disorders and/or women with disturbed circadian rhythms for other reasons.

## Introduction

Diverse and compelling evidence indicates that a profound connection exists between the circadian and reproductive systems at multiple levels of organization and at multiple stages of the reproductive cycle in mammals. Studies in rodents have demonstrated a critical role for the circadian clock in gating the timing of the pro-estrous LH surge and ovulation: both ablation of the master circadian clock in the suprachiasmatic nucleus (SCN) of the hypothalamus and disruption of the neural connections between the SCN and preoptic area eliminate estrous cyclicity and cause infertility in rats [1]. The circadian clock also coordinates seasonal cycles of reproduction in many mammalian species by relaying information regarding day length to neuroendocrine systems regulating reproductive function [2]. In humans, one study found that half of shift-working nurses experience changes in menstrual function [3]. Interestingly, three women involved in the study who were attempting to conceive reported infertility during shift work [3]. In addition, several epidemiological studies have observed associations between shift work or repeated travel across time zones and reduced fertility [4] as well as negative pregnancy outcomes, including increased incidence of low birth weight, preterm birth and miscarriage [5], [6], [7], although whether these adverse outcomes are due to circadian dysregulation or some other lifestyle factor associated with shift work is not known. For example, sleep loss is common in shift workers and a recent study suggests that poor sleep quality is associated with pre-term birth [8].

Studies in animal models have demonstrated that genetic disruption of the circadian clock negatively influences reproductive function and pregnancy outcomes. Mice with a mutation in the core circadian clock gene *Clock* have multiple reproductive abnormalities, including extended and irregular estrous cycles, increased fetal reabsorption during pregnancy and an elevated rate of full-term pregnancy failure [9], [10]. Mice with a null mutation of *Bmal1*, which encodes the binding partner of *Clock*, also display marked disruption of reproductive function, including infertility in males as well as irregular estrous cycles and an inability to carry pregnancy to term in females [1], [11]. Perinatal delivery problems and limited survival of pups to weaning have also been reported in *Vipr2^−/−^* mice [10], which have a null mutation of the *VPAC_2_ receptor* gene. This receptor is involved in photic entrainment of the SCN to the light:dark (LD) cycle [12], and *Vipr2^−/−^* mice have been shown to have defective SCN rhythms [13].

In contrast to genetic disruption, which may be complicated by pleiotropic effects of specific mutations, the effects of environmentally-imposed disturbances of the circadian system on pregnancy in mammalian model systems are less well understood, even though environmental disruptions may be more comparable to shift-workers and others that experience disrupted circadian organization in spite of an intact and fully functional circadian clock. The circadian clock is clearly implicated in the timing of estrous cycles and ovulation [14], as well as birth [15], but its role in gestation has not been carefully examined. Given the absence of comprehensive data on the effects of environmental disturbances of the circadian clock and pregnancy outcomes, we sought to determine the impact of repeated 6-hour phase shifts in the entraining LD cycle on reproductive success in the common laboratory mouse strain C57BL/6J (B6 hereafter). We observed that repeated shifts of the LD cycle substantially reduced reproductive success in mice, with phase advances having a greater impact than phase delays.

## Results

### The Impact of Circadian Disruption on Pregnancy Outcomes

**Figure 1:**
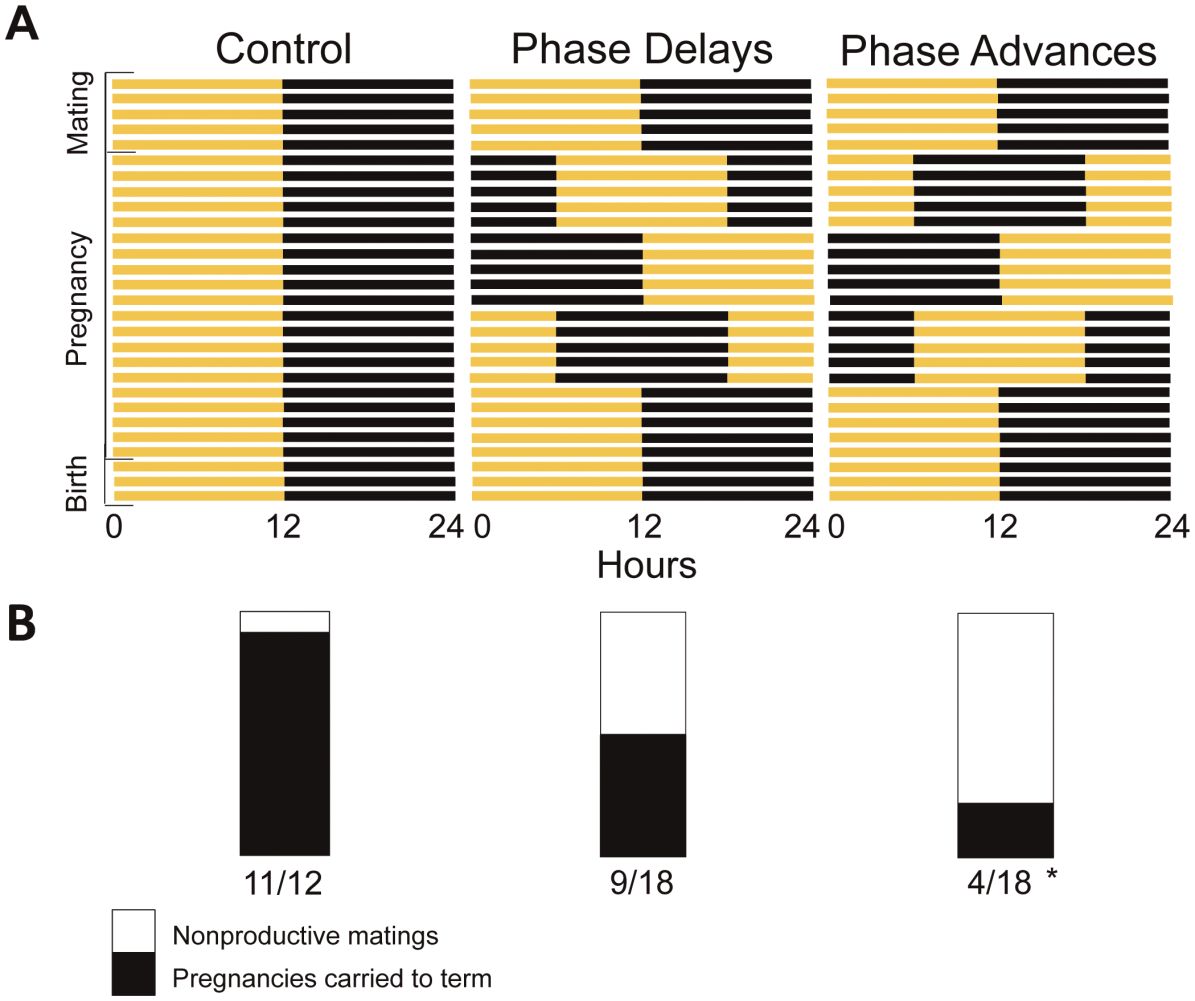
Phase delays or advances after copulation reduce the proportion of pregnancies carried to term. (A) After copulation was verified via identification of vaginal plugs, mice were randomized into control (*n* = 12), phase-delay (*n* = 18) or phase-advance groups (*n* = 18). Zero to four days after copulation, mice were transferred to new light-tight cabinets, each with 12-hr light and 12-hr dark light cycles, but differing in the time of light onset and offset. Control females remained in this chamber on a constant 12∶12 light (yellow bar):dark (black bar) cycle that matched the preceding one during mating, whereas females in the experimental groups were exposed to either 6-hour delays or advances in the light cycle, which was repeated by switching cabinets every 5–6 days for the duration of gestation. (B) The number of copulations successfully carried to term in each of the groups was recorded via daily visual inspection. Data comparisons were made using Pearson’s chi-square test (Phase delays: χ^2^ = 3.41, *P*<0.1; * Phase advances: χ^2^ = 9.47, *P*<0.01).

Young adult female B6 mice were allowed to mate with males. After copulation, which was verified by vaginal plug formation, female mice were randomized into one of three groups: control, phase-delay or phase-advance. The control group was undisturbed for the duration of pregnancy whereas the other groups were subjected to either 6-hour delays or advances of the light cycle every 5–6 days (Fig. 1a). After the fourth phase shift, which immediately preceded the expected litter birth date, the resulting light cycle was held constant. Reductions in pregnancies carried to term were observed in both of the phase shift groups, with a particularly significant decline in the phase-advanced females (Fig. 1b). Under normal LD conditions for the control mice, copulation led to full-term pregnancy over 90% of the time (*n* = 12). However, that success rate fell to 50% with exposure to phase delays after copulation (*n* = 18) and 22% with exposure to phase advances (*n* = 18).

These rates of pregnancy success were analyzed using Pearson’s chi-square test: the frequency of successful pregnancies carried to term in the control group (11/12) was used to determine the expected number of successful pregnancies in the experimental groups. The experimentally observed success rates (9/18 and 4/18 for phase-delay and advance groups, respectively) were then used to calculate the chi square values and p values.

### Re-entrainment of Locomotor Activity Rhythms After Phase Delays and Phase Advances

A possible explanation for the particularly adverse effects of phase advances in the LD cycle is that advances are more disruptive to the circadian system than delays, as indicated in a study reporting greater mortality in aged mice subjected to advances of the LD cycle than those subjected to delays [16]. This hypothesis is also supported by phase response curve (PRC) data indicating that the circadian system in mice is more capable of generating delays than advances in response to entraining light pulses [17], [18], [19].

**Figure 2:**
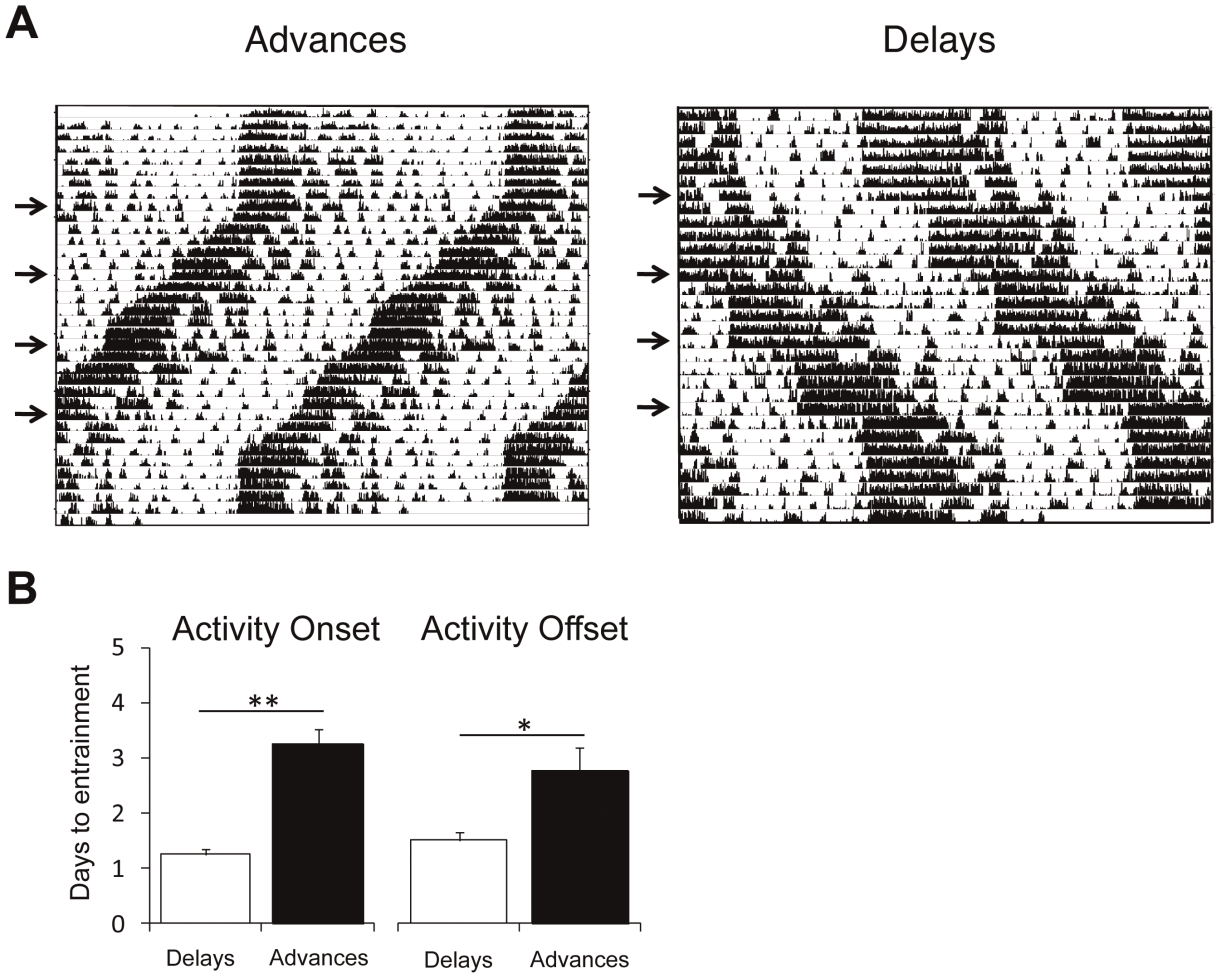
Mice entrain more rapidly after phase delays than phase advances. (A) Representative double-plotted actigrams from individually housed C57BL/6J females subjected to 6-hour phase advances (left) and phase delays (right) every 5–6 days. Time (48-hours) is shown on the x-axis and days are ordered sequentially down the y-axis. Each black tick mark indicates the break of an infrared beam within the animal’s cage. Arrows denote days when phase shifts occurred. (B) The number of days (mean ± s.e.m.) required for re-entrainment after a phase shift (for each group, *n* = 20 (5 animals×4 shifts)). Mice exposed to phase delays achieved entrainment to the new light cycle more rapidly than those exposed to phase advances (** *P*<0.001, * *P*<0.005; Student’s t-test).

Indeed, in a separate cohort of B6 females, we observed that a longer period of time was necessary for re-entrainment after phase advances than delays (Fig. 2). Furthermore, a study of rotating male shift-workers found that switching from an advancing to a delaying shift schedule led to improvements in worker satisfaction, productivity and subjective health reports [20], suggesting that phase delays are less physiologically disruptive than advances in humans as well as mice.

## Discussion

These results are consistent with an important role for synchronization between internal circadian organization and the external LD cycle in mediating successful pregnancy outcome. Females subjected to phase advances experienced a significant reduction in the proportion of matings resulting in pregnancies carried to term. Matings were classified as nonproductive if there was no indication of pregnancy as determined by gentle palpation on gestational day 18 and no evidence of subsequent births, suggesting that pregnancy loss occurred early in gestation and was not due to an impact of the phase shifts on delivery or post-partum litter maintenance.

Although a previous study in pregnant rats subjected to 12-hour phase reversals every 3–4 days failed to observe any adverse pregnancy outcomes [21], this may be due to the possibility that such frequent and dramatic changes in the light cycle are beyond the limits of entrainment, thus causing the circadian clock to run independently of the light cycle, as has been observed in rodents under non-circadian entrainment conditions [22]. Activity records of the mice in the study described here indicate that the 6-hour phase shift protocol does indeed result in repeated attempts at entrainment, indicating that the circadian system is continuously adapting to the changing light cycle (Fig. 2a).

These findings suggest that abnormalities in circadian entrainment underlie the observed effects on pregnancy outcomes, a hypothesis that has theoretical support from the literature. Early work in hamsters demonstrated that lesions of the maternal SCN on the seventh day of gestation caused desynchronized activity rhythms in pups after birth, indicating that maternal circadian rhythms participate in the entrainment and regulation of fetal rhythms early in gestation [23]. More recently, studies in rats and mice have shown that core circadian clock genes are expressed rhythmically in the gravid uterus and placental tissue, suggesting that circadian clocks in peripheral parturition-relevant organs contribute to the regulation of rhythms in the developing fetus [24], [25]. Shifts of the LD cycle in rats are known to transiently disrupt normal phase relationships between the SCN and peripheral oscillators [26], thus desynchronized circadian rhythms, either between central and peripheral maternal tissues or between maternal and fetal tissues (or both), may in part contribute to the adverse effects of chronic environmentally-mediated circadian disruption on pregnancy outcomes, although this hypothesis requires experimental verification.

Our results complement previous studies examining fertility and reproductive physiology in genetic models of circadian disruption, which have shown that delivery and peri- and post-natal development are hindered by the lack of an intact circadian clock [9], [10]. Here we show that even wild-type mice experience substantial reductions in pregnancy success when circadian entrainment is disturbed, and the congruence in findings between genetic and environmental models strongly implicates dysfunction of the circadian clock in the adverse developmental and reproductive phenotypes, as opposed to pleiotropic or non-circadian effects of specific mutations. Importantly, this animal model of environmental circadian disruption may have significant implications for the impact of circadian misalignment on reproductive function in humans, who often work or socialize out of synchrony with the external light cycle. Given the epidemiological linkages between shift work and repeated travel across time zones with reduced fertility and worsened pregnancy outcomes, unraveling the mechanisms linking circadian synchronization, reproductive physiology and pregnancy at the basic science level in animal models may provide insight into the contribution of disrupted circadian rhythms to miscarriage, premature birth and fertility problems, a potential risk factor poorly studied in humans.

## Materials and Methods

### Ethics Statement

All mice were housed and handled in accordance with Federal animal welfare guidelines and in compliance with the PHS Policy on Humane Care and Use of Laboratory Animals (2002) and the Guide for the Care and Use of Laboratory Animals (8^th^ Edition). Experiments were reviewed and approved prior to being carried out by the Northwestern University Institutional Animal Care and Use Committee (Animal Study Protocol# 2008-1412).

### Breeding and Phase-shift Experiments

Young adult male and female C57BL/6J mice were obtained from the Jackson Laboratory (Bar Harbor, ME). All animals were initially maintained on a 12-hour light:12-hour dark (12∶12 LD) cycle with lights on at 5 AM and lights off at 5 PM Central Standard Time. Animals were housed in ventilated, light-tight cabinets equipped with 40W GE “Cool White” fluorescent bulbs individually controlled by an electronic timer (Tork). After a minimum 1-week acclimation period prior to the experiment in which males and females were group-housed in single-sex cages, males and females were paired. Mating during the preceding night was determined by inspecting females for the presence of a vaginal (copulatory) plug [27]. After confirmation of copulation, each female was separated into an individual cage and randomly assigned to one of three groups: control, phase-delay or phase-advance. Zero to four days after mating, females’ cages were transferred to a separate cabinet that had one of three light cycles: the same phase (Lights on at 5 AM; Control group), a 6-hour delayed cycle (Lights on at 11 AM, Lights off at 11 PM; Phase-delay group) or a 6-hour advanced cycle (Lights on at 11 PM, Lights off at 11 PM; Phase-advance group). Females in the control group were then left in the same cabinet with a constant 12∶12 LD cycle for the remainder of the experiment, while females in the phase-shifted groups had their cages moved to a new cabinet and light cycle every 5–6 days. Females in the phase-delay group experienced a 6-hour delay every 5–6 days for three additional LD transfers (the phase shift was always achieved by manipulating light onset, which occurred on either day 5 or day 6). To achieve the phase-delay, the dark period was extended for 6 hours, and then a 12∶12 LD cycle was resumed for 5–6 days before the next shift. Females in the phase-advance group experienced a total of four 6-hour phase advances. To achieve the phase-advance, the light period was advanced by 6 hours, and then a 12∶12 LD cycle was resumed for the next 5–6 days. The protocol was designed to last for the duration of pregnancy (20–21 days). After the fourth phase shift, all animals remained in a constant 12∶12 LD cycle.

### Measurement of Pregnancy Outcomes

Females entered the experiment once copulation was verified via observation of the presence of a vaginal plug in the morning. Animals were visually monitored daily for the expected duration of pregnancy (20–21 days), as well as for at least an additional week past the expected litter birth date. While phase-shifted groups were in the reverse LD cycle (i.e., Lights on at 5 PM and Lights off at 5 AM), the daily checks were accomplished with the use of an infrared viewer (FJW Industries, Palatine, IL). Pregnancies carried to full-term and resulting in births of pups were scored as successful. Females were gently palpated to determine pregnancy status at approximately gestational day 18 (following the final light cycle shift). Females that were not found to be pregnant in this manner, and for whom no signs of birth were subsequently observed, were scored as nonproductive. Although no differences in litter size or pregnancy duration were apparent between any of the groups, we were not always able to determine the total number of pups because litters or parts of litters may have been destroyed by mothers shortly after birth.

### Measurement of Locomotor Activity

Virgin females were individually housed in cages outfitted for measurement of locomotor activity via infrared beam-breaks. Briefly, three infrared beams are projected across the cage and locomotor activity is recorded when any one of these beams is broken. Phase advance (*n* = 5) and phase delay (*n* = 5) groups were subjected to the same protocol used in the phase-shift experiments as described above. The time to entrainment was calculated as the number of days required for each animal to align locomotor activity (onset and offset were examined separately) to the new LD cycle.

### Statistical Analysis

Reproductive success rates were analyzed using Pearson’s chi-square test. The frequency of successful pregnancies carried to term in the control group (11/12) was used to determine the expected number of successful pregnancies in the experimental groups. The experimentally observed pregnancy success rates (9/18 and 4/18 for phase-delay and advance groups, respectively) were then used to calculate the test statistics, which were compared to a chi-square distribution with one degree of freedom. The locomotor activity data used to measure the time required for entrainment after a phase shift were compared using Student’s t-test.
